# *Streptococcus canis* outbreak affecting farmed mink (*Neogale vison*) in Northwestern Greece

**DOI:** 10.1007/s11259-026-11371-5

**Published:** 2026-06-24

**Authors:** Anna Maria Iatrou, Konstantinos Papageorgiou, Aikaterini Stoikou, Georgios Delis, Dimitrios Papadopoulos, Eleni Vatzia, Spyridon K. Kritas, Evanthia Petridou

**Affiliations:** 1 School of Agricultural Sciences, Department of Agriculture, University of Western Macedonia, Florina, Greece; 2 Laboratory of Microbiology and Infectious Diseases, School of Veterinary Medicine, Faculty of Health Sciences, Aristotle University of Thessaloniki, Thessaloniki, Greece; 3 Laboratory of Pharmacology, School of Veterinary Medicine, Faculty of Health Sciences, Aristotle University of Thessaloniki, Thessaloniki, Greece

**Keywords:** Antimicrobial resistance, Infection, *S.canis*, Encephalitis

## Abstract

The present study describes an outbreak of *Streptococcus canis (S.canis)* infection in a commercial mink farm in West Macedonia, Greece, and investigates its clinical, pathological and microbiological features.

In late February 2024, approximately 300 out of 5,100 mink (5.9%) developed purulent abscesses primarily located on the head, while some animals exhibited neurological signs, including depression, weakness and lethargy. The farmer reported similar incidences in animals also the previous year. Necropsy revealed localized abscessation, and purulent meningoencephalitis was observed in two cases. Bacteriological culture followed by Oxford Nanopore Technologies sequencing confirmed the presence of *S. canis* in all examined samples. Antimicrobial susceptibility testing demonstrated potential clinical efficacy of ampicillin and erythromycin.

The recurrence of the outbreak and its seasonal pattern suggest the involvement of predisposing factors such as environmental stress and trauma associated with housing conditions. Clinical response to ampicillin supports its continued efficacy against *S.canis*, while resistance to selected non-β-lactam agents highlights the value of antimicrobial susceptibility testing when alternative therapies are considered.

These findings support a potential pathogenic role of *S.canis* in farmed mink, including cases with neurological involvement, and underline the need for improved management practices and further investigation into its epidemiology in farmed mink populations.

## Introduction

*Streptococcus canis (S.canis)* is a Gram-positive, β-hemolytic, Lancefield serogroup G, multi-host bacterium isolated from a broad range of mammals and widely recognized as an opportunistic pathogen in carnivores (Olson 1975; Corning et al. 1991; Devriese 1991; Iglauer et al. 1991; Bornand et al. 1992). In domestic animals, such as dogs and cats, it is usually found on mucosal membranes and skin; however, it has also been associated with a wide spectrum of other clinical conditions, including dermatitis, septicaemia, pneumonia, and neonatal mortality (Lyskova et al. 2007; Lamm et al. 2010).

Beyond companion animals, *S.canis* has also been reported in wildlife and livestock, where it can persist as part of the commensal microbiota or emerge as a primary pathogen when host immunity is compromised or environmental stressors are present. Although subclinical mastitis by serogroup G streptococci is rare, with a reported incidence of < 1%, *S.canis* has been described as a contagious cause with cow-to-cow transmission (Watts et al. 1984; Fox and Gay 1993; Soedarmanto et al. 1996; Hassan et al. 2005).

In addition, *S.canis* has been sporadically isolated from humans, particularly among individuals with exposure to companion animals (Bert and Lambert-Zechovsky 1997; Takeda et al. 2001; Whatmore et al. 2001; Taniyama et al. 2017; Zaidi and Eranki 2019; Lederman et al. 2020). Historically, some infections may have been identified only as “Group G Streptococcus” based on Lancefield typing, potentially leading to underrecognition of *S.canis* prior to the widespread use of MALDI-TOF MS and molecular identification methods. Reported human infections may result in severe clinical manifestations such as septicaemia and endocarditis (Lacave et al. 2016; Malisova et al. 2019). These findings underscore the bacterium’s zoonotic potential and suggest that close animal–human interactions may facilitate transmission under certain conditions (Amsallem et al. 2014; Pagnossin et al. 2023).

*Streptococcus* spp. constitute part of the commensal microbiota in many tissues and have been isolated from samples including heart, skin lesions, lungs, brain and intestines (Crawford et al. 2025). In mink, several *Streptococcus* species, including *S.canis*, *S.phoacae*, *S*.*lutetiensis* and *S.halichoeri* have been previously reported (Eklund et al. 2020; Nikolaisen et al. 2021; Crawford et al. 2025). Under appropriate conditions, however, they may assume an opportunistic role, giving rise to clinical manifestations of variable severity, ranging from superficial cutaneous lesions to more extensive infections. Moreover, in mink, *S.canis* has been repeatedly associated with outbreaks of pododermatitis (Chalmers et al. 2015; Brojer 2000). This broad tissue distribution emphasizes the potential for both localized and systemic disease in affected animals.

Although *S.canis* is relatively well-studied in companion animals, knowledge of its epidemiology and pathogenicity in farmed mink remains limited. This gap is of particular concern given the intensive nature of mink farming, where high animal density and environmental stressors can facilitate the emergence and spread of opportunistic pathogens. Furthermore, while other *Streptococcus* species have been extensively documented in mink, there are few detailed reports of *S.canis* infections in this species.

The present study describes an outbreak of *S.canis* infection in farmed mink in the region of West Macedonia, Northwestern Greece. It presents the clinical and pathological findings and discusses the potential predisposing factors and management implications which may facilitate disease occurrence. To the authors’ knowledge, this is one of the few reports describing *S.canis*-associated disease with neurological symptoms in farmed mink.

## Materials and methods

In 2024, an outbreak was recorded in a mink farm in Kastoria, West Macedonia, Greece, housing 5,100 animals where approximately 300 mink (5.9%) developed purulent lesions characterized by abscesses mainly on the head, while some of the affected animals showed neurological symptoms including depression, weakness and lethargy. Animals were bred under a normal intensive production system, being housed individually in wire mesh cages, provided with straw bedding material. Specifically, wheat straw was provided throughout the winter and reproductive period, as is common practice in mink farming. Animals were fed a commercial mink diet. No recent introductions of new breeding stock or major alterations in management practices were reported prior to the outbreak. The lesions and clinical signs were observed mainly in February and sporadically in March. According to the farmer, a similar issue had occurred the previous year, but no further investigation was performed. The cases showed a partial response to amoxicillin administration; however, clinical signs reappeared shortly after treatment cessation.

**Fig. 1:**
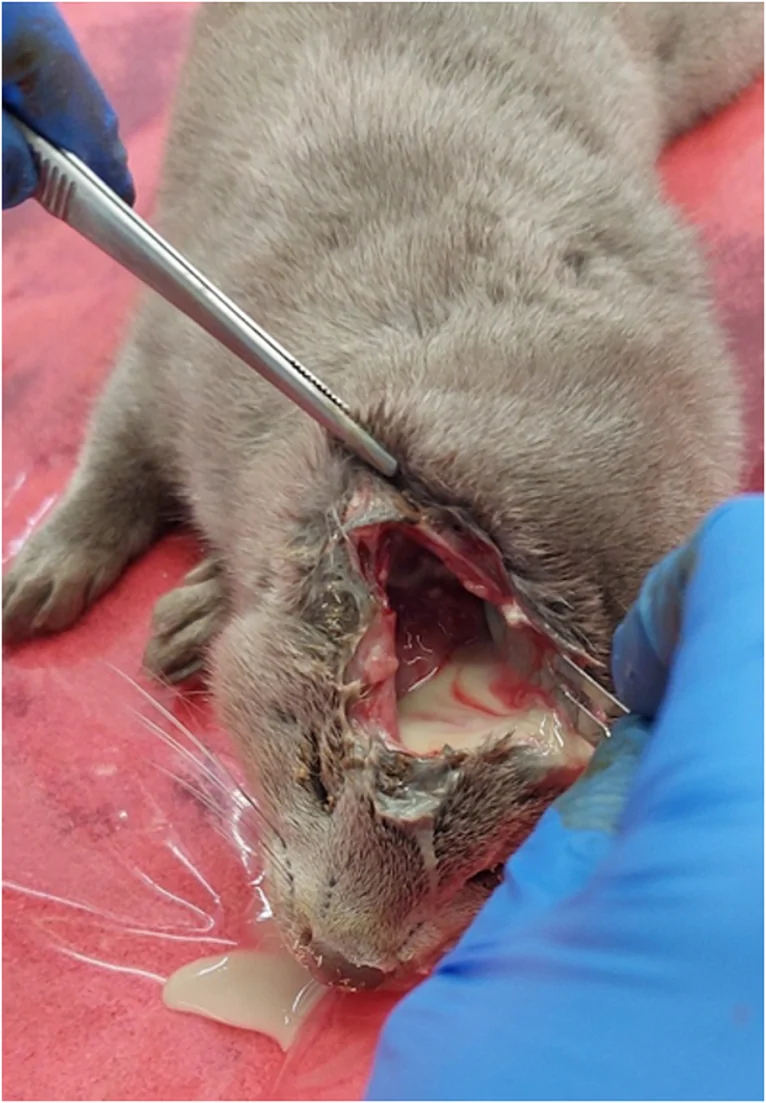
Purulent abscess in the head region of an affected mink. The lesion extended intracranially and was associated with purulent meningoencephalitis observed during necropsy. This photograph was obtained during necropsy examination of an animal from the present outbreak

Full necropsies were performed on the farm on 10 animals, revealing lesions confined to the head. Specifically, upon incision of the abscesses, serohaemorrhagic exudate and purulent material were observed. An additional 3 deceased mink were submitted to the Laboratory of Microbiology and Infectious Diseases, School of Veterinary Medicine, Aristotle University of Thessaloniki, for further diagnostic investigation. In two of the three minks, purulent meningoencephalitis was evident during necropsy (Fig. 1). Therefore, brain tissue samples were collected from all submitted animals and were analysed using conventional microbiological techniques and Oxford Nanopore Technologies (ONT) sequencing.

Samples were cultured on Blood Agar (Oxoid, Hampshire, UK) and MacConkey agar plates (Oxoid, Hampshire, UK) and incubated at 37 ^ο^C overnight. For the isolation of anaerobic bacteria, the same procedure was followed, with plates incubated under anaerobic conditions at 37 ^ο^C for 48 h.

DNA extraction of the isolated colonies on Blood agar was performed using the Nucleospin Microbial kit (Macherey-Nagel, Duren, Germany). In addition, DNA was extracted directly from brain tissue of the submitted mink using the DNeasy Blood and Tissue kit (Qiagen, Hilden, Germany). Subsequently, the extracted DNA was subjected to Oxford Nanopore Technologies (ONT) sequencing using the Rapid Barcoding Kit 24 V14 SQK-RBK114.24 (Oxford Nanopore Technologies, Oxford, UK). The prepared libraries were loaded into a R10.4.1 flow cell (Oxford Nanopore Technologies, Oxford, UK) and sequenced using the MinION Mk1B device (Oxford Nanopore Technologies, Oxford, UK). Raw data were basecalled with Guppy v6.5.7 and analysed using the EPI2ME platform (Oxford Nanopore Technologies, Oxford, UK). Specifically, the EPI2ME metagenomic workflow was used for taxonomic classification and species-level identification of bacterial DNA present in the samples. No whole-genome assembly or further genomic characterization analyses were performed.

Antimicrobial susceptibility testing of the isolated bacteria was performed by disk diffusion following EUCAST recommendations. Since no veterinary-specific breakpoints for *S.canis* are currently available in EUCAST v16.0 guidelines, interpretation was based on the available human *Streptococcus* spp. breakpoints according to EUCAST v16.0 guidance.

No experimental procedures were performed. Samples were collected as part of routine diagnostic investigation.

## Results and discussion

After incubation, small greyish-white colonies with β-haemolysis were observed on blood agar of all the samples. The isolated colonies were determined to be catalase negative Gram-positive cocci consistent with *Streptococcus* spp. Analysis of ONT sequencing data using the EPI2ME metagenomic workflow identified *S.canis* DNA both in bacterial isolates recovered from culture and directly in the corresponding brain tissue samples, with concordant results across all examined cases, supporting the bacteriological findings and suggesting a possible association with the observed clinical syndrome. The antimicrobial susceptibility of the isolates is shown in Table 1.

**Table 1:** Antimicrobial agent susceptibilities of *S.canis* (*Streptococcus* spp. Group G)

Antimicrobial agent	Disk content	Zone diameter (mm)	Interpretation
Penicillin G	1 U	28	Susceptible
Erythromycin	15 µg	24	Susceptible
Tetracycline	30 µg	15	Resistant
Clindamycin	2 µg	15	Resistant

These results refer to bacteria isolated from all examined samples. More specifically, three isolates, corresponding to one isolate from each examined brain sample, were tested and all demonstrated identical susceptibility profiles. According to the data presented in Table 1, the isolates were susceptible to Penicillin G and erythromycin, while resistance to tetracycline and clindamycin was observed.

The present study supports a potential pathogenic role of *S.canis* in farmed mink and expands the limited knowledge regarding streptococcal infections in this species. Although *S.canis* is commonly regarded as part of the commensal flora of carnivores, its isolation in the affected animals indicates that it may play a pathogenic role under certain circumstances. The observed clinical improvement following β-lactam antimicrobial administration further supports the potential involvement of *S.canis* in the disease process, although the possibility of concurrent undetected microorganisms cannot be completely excluded. This finding is consistent with previous reports describing the opportunistic nature of *S.canis* in dogs and cats, where it may cause localized abscesses, dermatitis, or systemic disease when host defenses are compromised (Lyskova et al. 2007; Lamm et al. 2010).

The recurrence of the case over two consecutive years suggests that specific environmental or host-related factors may have contributed to disease emergence. Notably, the seasonal pattern, mainly in February and early March, coincides with a period of increased stress for the animals due to low temperatures and possibly because of dietary changes typically implemented before mating. Such stressors are known to modulate immune responses, predisposing animals to opportunistic infections (Earley et al. 2010). During this period, animals have permanent access to straw, which they often bite or ingest, occasionally resulting in injuries to the oral cavity. Combined with their excitable and nervous behavior, this likely increases the incidence of oral or facial trauma. Straw-associated oral trauma and the possible presence of foreign material in the oral cavity were therefore considered plausible predisposing factors facilitating bacterial invasion and local inflammation. This hypothesis is consistent with previous observations that mucosal trauma can serve as an entry point for *S.canis* and other opportunistic streptococci (Chalmers et al. 2015; Brojer 2000). However, *S.canis* may represent either a primary pathogen or a secondary invader in the observed lesions, and its exact role in disease pathogenesis cannot be definitively established based on the present findings. The multifactorial nature of streptococcal infections in farmed mink should also be considered. Intensive housing conditions, close animal proximity, repeated environmental exposure and stress-associated behavioral patterns may collectively facilitate bacterial persistence and transmission within the farm environment. Similar management-associated risk factors have been implicated in outbreaks involving *Streptococcus* spp. and other opportunistic bacterial pathogens in farmed mink populations (Chalmers et al. 2015; Crawford et al. 2025).

The absence of histopathological examination further limits interpretation of lesion pathogenesis, differentiation between primary and secondary infection, and assessment of possible mixed infectious processes. In addition, the ONT sequencing approach used in the present study was limited to taxonomic identification through the EPI2ME metagenomic workflow. Therefore, detailed genomic analyses, including genome assembly, multilocus sequence typing (MLST), antimicrobial resistance gene characterization, virulence-associated gene profiling, and comparative genomic analyses, were not performed. Further studies incorporating whole-genome sequencing would be valuable for improved epidemiological characterization of *S.canis* isolates from farmed mink.

Based on the results of antimicrobial susceptibility testing the farmer adopted ampicillin as the antimicrobial of choice. The favorable clinical response to ampicillin supports its continued efficacy against *S.canis*, consistent with previous reports describing β-lactams as the drugs of choice for streptococcal infections in animals (Tikofsky and Zadoks 2005). Nevertheless, the detection of resistance to selected non-β-lactam antimicrobial agents, including tetracycline and clindamycin, highlights the value of antimicrobial susceptibility testing when alternative therapeutic options are considered. Previous studies from farmed mink in Denmark reported high levels of resistance to tetracycline and erythromycin among *S.canis* isolates, while resistance to penicillin remained low (Nikolaisen et al. 2017). In the present study, the isolates remained susceptible to erythromycin, indicating possible differences in local antimicrobial resistance patterns between mink populations. According to the farmer, no additional antimicrobial agents beyond those described in the present study had been used during the outbreak period. The previously observed partial response to amoxicillin treatment on the farm may have been influenced by limited penetration of the antimicrobial into the central nervous system in animals already exhibiting neurological involvement. Based on the observed susceptibility to Penicillin G, susceptibility to other β-lactam antimicrobials, including cephalosporins, may generally be inferred according to current EUCAST and CLSI guidance for *Streptococcus* spp.

Furthermore, in addition to treatment with ampicillin, replacement of the straw with a softer alternative was suggested to the farmer. The recurrence of the disease over two consecutive years may suggest the involvement of persistent management, environmental, or host-related predisposing factors. Increased nervousness and excitable behaviour observed in some animals may have contributed to a higher risk of trauma and subsequent infection. Therefore, exclusion of severely affected or particularly aggressive animals from breeding was suggested as a precautionary management measure. For this reason, it was recommended that neither affected animals nor their offspring be retained as breeding stock for the next reproductive season. In addition, animals showing increased nervousness, as well as their descendants, were recommended to be excluded from the breeding population as a precautionary management measure (Pedersen et al. 2004).

In conclusion, streptococcal infection in farmed mink represents a multifaceted challenge involving pathogen behavior, antimicrobial resistance and farm management practices. This outbreak underscores the importance of prompt diagnosis and targeted antimicrobial treatment. Understanding the clinical and pathological manifestations of *S.canis* is essential for improving diagnostic accuracy and farm management practices. Although occasional human infections caused by *S.canis* have been reported, particularly following close animal contact, the present study did not investigate zoonotic transmission or comparative genomic relationships between animal and human isolates. Therefore, the zoonotic significance of mink-associated strains remains unclear and needs further investigation.

## Data Availability

The datasets generated during and/or analysed during the current study are available from the corresponding author on reasonable request.
